# Regulation of the prometastatic neuregulin–MMP13 axis by SRC family kinases: therapeutic implications

**DOI:** 10.1002/1878-0261.12145

**Published:** 2017-10-31

**Authors:** Ana Orive‐Ramos, Samuel Seoane, Alberto Ocaña, Atanasio Pandiella, Juan Carlos Montero

**Affiliations:** ^1^ Instituto de Biología Molecular y Celular del Cáncer (IBMCC‐CIC) CSIC Salamanca Spain; ^2^ Unidad de Investigación Traslacional Hospital Universitario de Albacete Universidad de Castilla La Mancha Albacete Spain; ^3^ Centro Regional de Investigaciones Biomédicas (CRIB) Universidad de Castilla La Mancha Albacete Spain; ^4^ CIBERONC Madrid Spain

**Keywords:** breast cancer dissemination, dasatinib, MMP13, neuregulin, SRC

## Abstract

Metastatic dissemination of tumor cells is responsible for the fatal outcome of breast cancer. Therefore, understanding the mechanisms involved in dissemination is essential for the development of new therapeutic strategies to prevent metastasis. One mechanism involved in metastatic dissemination of breast cancer cells is dependent on control of the production of matrix metalloproteinases by the neuregulins (NRGs). The NRGs are polypeptide factors that act by binding to the ErbB/HER subfamily of receptor tyrosine kinases. NRG‐mediated activation of HER receptors causes an increase in the production of metalloprotease 13 (MMP13, also termed collagenase‐3), which facilitates metastatic dissemination of breast tumors. In this context, we aimed to explore whether the clinically approved tyrosine kinase inhibitor dasatinib was able to neutralize this mechanism of metastatic dissemination. Here, we show that dasatinib restricted NRG‐induced MMP13 upregulation, both *in vitro* and *in vivo*, and *in vivo* metastatic dissemination of breast cancer cells. Chemical proteomics studies showed that the main cellular targets of dasatinib were SRC family kinases (SFKs). Moreover, genetic studies showed that knockdown of SRC or YES strongly inhibited NRG‐induced MMP13 upregulation *in vitro*. Mechanistically, dasatinib treatment or knockdown of SRC also inhibited ERK1/2 kinases *in vitro*, which were required for NRG‐induced MMP13 upregulation. These results open the possibility of clinically exploring the antitumoral action of dasatinib in those tumors in which the NRG–MMP13 signaling axis may play a relevant role in the control of tumor cell dissemination.

**Abbreviations**
Luc: luciferase
MMP13: matrix metalloproteinase 13
NRGs: neuregulins
SFKs: SRC family kinases

## 1 Introduction

The neuregulins (NRGs), a group of polypeptides that belong to the epidermal growth factor family of ligands (Montero *et al*., 2008), were initially identified while searching for ligands able to activate the HER2 transmembrane tyrosine kinase (Holmes *et al*., 1992; Peles *et al*., 1992). The interest in the identification of activators of HER2 was promoted by the finding that this membrane protein was overexpressed in a subset of patients with breast cancer, and such overexpression correlated with metastatic dissemination of the disease and poor patient outcome (Slamon *et al*., 1987).

While NRGs were initially isolated by their ability to activate HER2, they do not bind to HER2 (Peles *et al*., 1993; Sliwkowski *et al*., 1994). Instead, they promote activation of HER2 indirectly, by first binding to other HER family members, such as HER3 or HER4 (Carraway *et al*., 1995; Tzahar *et al*., 1994). Four different NRG genes (*NRG1‐4*) that code for 32 distinct isoforms have been described (Esparís‐Ogando *et al*., 2016; Falls, 2003; Montero *et al*., 2008). NRG1 and NRG2 isoforms bind to HER3 and HER4 receptors (Carraway *et al*., 1997; Falls, 2003; Hynes and Lane, 2005), while NRG3 and NRG4 isoforms bind to HER4 (Hobbs *et al*., 2002). Upon binding to HER3 or HER4, these receptors undergo a conformational change exposing the so‐called dimerization arm, located in the subdomain II of their extracellular regions (Burgess *et al*., 2003). Exposed dimerization arms allow two receptors to dimerize, starting signal propagation (Esparís‐Ogando *et al*., 2016; Sánchez‐Martín and Pandiella, 2012). While in the case of HER3 and HER4 ligand binding is required to expose the dimerization arm, that region is already available in the case of HER2 in the absence of any ligand binding (Cho *et al*., 2003). Because of that, HER2 is predisposed to interact with other HER receptors (Graus‐Porta *et al*., 1997).

Several clinicopathological and preclinical studies have implicated the NRGs in the pathophysiology of breast cancer. In fact, NRG expression has been detected in a substantial proportion of tumors from patients with breast cancer, especially in tumors with normal levels of HER2 (Montero *et al*., 2008). Modeling of that situation *in vitro* by inducing NRG expression in breast cancer cells that bear normal levels of HER receptors resulted in the creation of a strong protumorigenic autocrine loop, characterized by increased proliferation (Yuste *et al*., 2005b). In these models, treatment with trastuzumab, a humanized monoclonal antibody that acts on the extracellular domain of HER2, prevented the proliferative responses due to NRG expression (Yuste *et al*., 2005b). This situation may have a clinical correlate, as expression of NRGs has been reported to biomark a subgroup of patients that may be sensitive to trastuzumab in the absence of HER2 amplification (de Alava *et al*., 2007; Paik *et al*., 2008).

Neuregulin expression has been linked to poor patient outcome in breast cancer, likely by facilitating tumor growth and metastatic dissemination of breast cancer cells (Seoane *et al*., 2016). Thus, clinicopathological analyses demonstrated that NRG expression was associated with dissemination of breast cancer cells to lymph nodes (Seoane *et al*., 2016). Mechanistic studies led to the identification of matrix metalloproteinase 13 [MMP13, also termed collagenase‐3 (Freije *et al*., 1994)] as an important mediator of metastatic dissemination in breast cancer cells in which the NRG–HER signaling axis was active (Seoane *et al*., 2016). Activation of HER signaling by expression of NRG in breast tumors led to the constitutive upregulation of the PI3K/AKT/mTOR and the RAS/RAF/ERK routes. Pharmacologic and genetic studies demonstrated that the latter route participated in the control of MMP13 production upon activation of HER receptors by NRGs. Such upregulation of MMP13 production was due to a transcriptional effect in which an SBF‐like transcription factor, controlled by the ERK pathway, regulated mRNA production responsible for MMP13 production (Seoane *et al*., 2016).

The identification of this mechanism of metastatic dissemination controlled by the NRG–HER–MMP13 signaling axis allows now to explore strategies to impede its functioning. Here, we show that dasatinib, a small tyrosine kinase inhibitor with intermediate selectivity (Karaman *et al*., 2008; Shah *et al*., 2004), restricts MMP13 upregulation and metastatic dissemination of breast cancer cells once the NRG–HER signaling axis is activated. Chemical proteomics, as well as genetic analyses, demonstrated that the effect of dasatinib was mediated by the SRC family of cytosolic kinases, which are among the known dasatinib targets (Karaman *et al*., 2008). These findings open the possibility of using this drug to impede dissemination of tumors fed by NRG or other factors that augment MMP13.

## 2 Materials and methods

### 2.1 Reagents and antibodies

Dulbecco's modified Eagle medium (DMEM), Roswell Park Memorial Institute (RPMI) 1640 medium, fetal bovine serum (FBS), penicillin, and streptomycin were purchased from Life Technologies (Carlsbad, CA, USA). Dasatinib was obtained from LC Laboratories (Woburn, MA, USA). Trametinib and BIX02189 were from SelleckChem (Houston, TX, USA). PP2 was from Calbiochem (San Diego, CA, USA). Neuregulin 1 β2 (NRG) was purchased from Prospec (Rehovot, Israel). Other generic chemicals were from Sigma‐Aldrich (St. Louis, MO, USA), USB Corporation (Cleveland, OH, USA), or Merck (Darmstadt, Germany).

Antibodies against MMP13, phospho‐Tyr (PY99), ERK1/2, and GAPDH were from Santa Cruz Biotechnology (Santa Cruz, CA, USA). Antibodies against phospho‐RAF (Ser259), phospho‐MEK1/2 (Ser217/221), phospho‐ERK1/2 (Thr202/Tyr204), phospho‐S6 (Ser240/244), phospho‐SRC family (Tyr416), SRC, CSK, YES, FYN, LYN, and LCK were from Cell Signaling Technologies (Beverly, MA, USA). The anti‐SHC antibody was purchased from Merck Millipore (Darmstadt, Germany). The anti‐phospho‐AKT (Ser473) antibody was from BD Biosciences (San Jose, CA, USA). The anti‐RAS antibody was kindly provided by Eugenio Santos (IBMCC, Salamanca, Spain). The 4D5 anti‐HER2 antibody used for immunoprecipitation was generously provided by Mark X. Sliwkowski (Genentech, South San Francisco, CA, USA). The anti‐HER2 (Ab‐3) antibody used for western blotting was from Calbiochem. The BCN4797 anti‐HER3 antibody was from AntibodyBcn (Barcelona, Spain). Antibodies against ERK5, phospho‐ERK5 (TEY), and NRG have been described previously (Esparís‐Ogando *et al*., 2002; Montero *et al*., 2000). Anti‐MEK5 and anti‐phospho‐MEK5 antibodies have been produced in our laboratory, and their generation will be described elsewhere. The horseradish peroxidase (HRP)‐conjugated antibodies: anti‐mouse, anti‐rabbit, and anti‐rabbit light chain specific were obtained from GE Healthcare Life Sciences (Piscataway, NJ, USA), Bio‐Rad Laboratories (Hercules, CA, USA), and Jackson ImmunoResearch Laboratories (West Grove, PA, USA), respectively.

### 2.2 Cell culture and cell treatment

All cell lines were cultured at 37 °C in a humidified atmosphere of 5% CO_2_ and 95% air. Cells were grown in DMEM containing high glucose concentration (4500 mg·L^−1^) or in RPMI 1640 medium, supplemented with antibiotics (100 U·mL^−1^ penicillin, 100 μg·mL^−1^ streptomycin) and 10% FBS. MCF7 cells expressing proNRGα2C (MCF7‐NRGα2C) and proNRGα2C‐Luciferase (MCF7‐NRGα2C‐Luc) have been previously described (Seoane *et al*., 2016; Yuste *et al*., 2005b). Where indicated, cells at 80% confluence were serum‐starved for 16–18 h and treated with 0.1% DMSO (vehicle control), 1 μm dasatinib (unless otherwise indicated), 1 μm trametinib, 10 μm BIX02189, or 10 μm PP2 for 3 h. Subsequently, cells were stimulated or not with 10 nm NRG for the indicated times and collected for protein extraction or RNA isolation.

### 2.3 Protein extraction, immunoprecipitation, and western blotting

Protein extraction, immunoprecipitation, and western blotting were performed as described previously (Seoane *et al*., 2010). GAPDH was used as a loading control. Densitometric measurements of the bands were taken using the public domain imagej program (NIH, Bethesda, MD, USA), and graphpad prism 6.0 (GraphPad Software, La Jolla, CA, USA) was used to determine IC_50_ values.

### 2.4 RNA isolation and qPCR

Total RNA from MCF7 cells was isolated using TRIzol^®^ reagent (Invitrogen, Carlsbad, CA, USA) according to the manufacturer's instructions. First‐strand cDNA was synthesized using M‐MLV reverse transcriptase and oligo‐dT (Invitrogen) following the instructions from the manufacturer. Quantitative retro‐transcriptase PCR (qRT‐PCR) assays were performed in duplicate in 96‐well optical plates on an iCycler^®^ iQ™5 thermal cycler (Bio‐Rad Laboratories) with SYBR Select Master Mix for CFX (Invitrogen) as described previously (Seoane *et al*., 2016). Levels of MMP13 mRNA were normalized against that of GAPDH and were relativized to those from unstimulated cells using the 2^−(ΔΔCt)^ method. The sequences of the primers (Invitrogen) used for gene expression analysis are as follows: MMP13 forward 5′‐TTGAGCTGGACTCATTGTCG‐3′, MMP13 reverse 5′‐CGCGAGATTTGTAGGATGGT‐3′, GAPDH forward 5′‐GAGTCAACGGATTTGGTCGT‐3′ and GAPDH reverse 5′‐GATCTCGCTCCTGGAAGATG‐3′. Results are presented as the mean ± SD of two independent experiments.

### 2.5 RAS‐GTP pull‐down

The RAS‐GTP levels were estimated by pull‐down assays using glutathione Sepharose 4B beads (GE Healthcare Life Sciences) previously coupled to the GST fusion protein of the RAS‐GTP‐binding domain from RAF (GST‐RBD) as previously described (Yuste *et al*., 2005a). Active RAS bound to the fusion protein was analyzed by western blotting using the specific anti‐RAS antibody.

### 2.6 Dasatinib immobilization and purification of its targets

For dasatinib immobilization, 5 mg of dasatinib was dissolved in a coupling solution composed of 50% dimethylformamide (DMF) and 50% 0.1 m Na_2_CO_3_, and was incubated with 0.5 mg of epoxy‐activated Sepharose 6B (GE Healthcare Life Sciences), previously washed with 100 mL of distilled water, in agitation for 16 h at room temperature. Uncoupled dasatinib was washed away with the coupling solution, and the remaining active groups were blocked with 0.1 m Tris/HCl pH 8.0 for at least 4 h at room temperature. Later, the dasatinib‐coupled resin was washed with at least three cycles of alternating pH (0.1 m acetate buffer pH 4.0 containing 0.5 m NaCl followed by a wash with 0.1 m Tris/HCl buffer pH 8.0 containing 0.5 m NaCl). Finally, the resin was resuspended in phosphate‐buffered saline (PBS) containing 0.05% sodium azide and stored at 4 °C. The control resin was prepared as described above but without dasatinib. To purify the different targets of dasatinib, 25 mg of MCF7 cells lysate protein was incubated with 100 μL of dasatinib‐containing resin or control resin for 3 h at 4 °C. Subsequently, complexes were precipitated by centrifugation and washed three times with lysis buffer (20 mm Tris/HCl pH 7.0, 140 mm NaCl, 50 mm EDTA, 10% glycerol, 1% Nonidet™ P‐40). Complexes were then resuspended and boiled in electrophoresis sample buffer and analyzed by SDS/PAGE (10% acrylamide/bisacrylamide concentration). The gel was silver‐stained, and bands of interest were excised from the gel and digested with trypsin. The peptide mass fingerprint was obtained on an Ultraflex MALDI‐TOF mass spectrometer (Bruker Daltonics, Bremen, Germany), and Mascot search engine (Matrix Science, London, UK) against Swiss‐Prot database was used to identify proteins. One result was considered to be significant (*P *<* *0.05) when the protein score value exceeded 56. To validate the different targets of dasatinib, 2 mg of MCF7 cell extract was incubated with 60 μL of dasatinib‐coupled resin or control resin for 3 h at 4 °C. Complexes were processed as described above and analyzed on SDS/PAGE followed by western blotting using specific antibodies against SFKs.

### 2.7 Lentivirus production and infection

For lentivirus production, 4 μg of the following plasmids: pMDLg/RRE, pRSV‐Rev and pMD2.G (Addgene, Cambridge, MA, USA), along with 8 μg of the pLKO.1 lentiviral plasmid containing a scramble shRNA (sh‐Control) or the indicated shRNA (GE Dharmacon, Lafayette, CO, USA), was cotransfected into HEK293T cells using jetPEI^®^ reagent (Polyplus‐transfection, Illkirch, France) following the manufacturer's instructions. 24 h later, HEK293T medium was replaced with fresh medium, and 48 h after the cotransfection, the medium containing lentiviral particles was collected, filtered, and used to infect MCF7 cells after the addition of 6 μg·mL^−1^ polybrene (Sigma‐Aldrich). MCF7 cells were cultured for 48 h to allow for efficient protein knockdown and were subsequently selected with 3 μg·mL^−1^ puromycin (Sigma‐Aldrich) for another 48 h. A minimum of five different shRNA sequences targeting SRC, YES, or CSK were tested, and those two that produced higher knockdown were used. Representative results using a shRNA sequence are shown.

### 2.8 Cell proliferation, wound healing, and cell invasion assay

Cell proliferation was assessed by MTT metabolization as previously described (Seoane *et al*., 2010). For cell migration analysis, the wound healing assay was performed as described previously (Montero *et al*., 2011b). After NRG stimulation, images were captured every 2 h until the wound in NRG‐stimulated cells was healed or for a maximum of 48 h with a Nikon Eclipse TE2000‐E inverted microscope (Nikon Corporation, Chiyoda‐ku, Tokyo, Japan) equipped with the metamorph^®^ Microscopy Automation and Image Analysis Software (Molecular Devices LLC, Sunnyvale, CA, USA). The area between the wound edges was measured using the imagej program (NIH, Bethesda, MD, USA) and relativized to the initial area. The cell invasion assay was performed as previously described (Montero *et al*., 2011b). The number of invading cells was counted under a Nikon Eclipse T*i*‐S inverted microscope (Nikon Corporation) using the progres ^®^ capturepro 2.7 program (Jenoptik AG, Jena, Germany). Results are presented as the mean ± SD of triplicate or images of a representative experiment that was repeated three times.

### 2.9 Xenograft studies

Mice were maintained and manipulated at the animal facility of the University of Salamanca following legal and institutional guidelines. Female BALB/c *nu*/*nu* mice (7 weeks old) were from Charles River Laboratories (Wilmington, MA, USA). MCF7‐NRGα2c‐Luc cells (5 × 10^6^) were injected into the mammary fat pad, at two sites per mice, as described previously (Seoane *et al*., 2016). Tumor diameters were serially measured with calipers every four days, and tumor volumes were calculated by the following formula: volume = (width^2 ^× length)/2. When tumors reached 100 mm^3^ (30 days after injection), mice were randomized into two groups (*n *=* *5 per experimental group) to receive orally either 25 mm tartaric acid (vehicle control) or 10 mg·kg^−1^ dasatinib once daily. Anesthetized mice were intraperitoneally injected with 100 mg·kg^−1^ D‐luciferin potassium salt (Regis Technologies, Morton Grove, IL, USA) 15 min before imaging, and bioluminescence images were acquired with an IVIS 50 Imaging System (Xenogen, Alameda, CA, USA) equipped with the living image software (Xenogen). Tumor sizes of primary tumors were measured weekly from the beginning of the treatment by photon flux emission. Local spreading was analyzed by measuring the width of the luminescent signals 90 days after injection. Data are presented as the mean ± SD of the primary tumors assayed in each experimental condition. Primary tumor samples obtained after killing the animals were processed as described previously (Seoane *et al*., 2016). The expression of MMP13, phospho‐SFKs, phospho‐ERK1/2, and ERK1/2 was analyzed by western blotting.

### 2.10 Statistical analyses

Data were analyzed statistically using the software package spss 15.0 (SPSS Inc., Chicago, IL, USA). Comparison of continuous variables between two groups for *in vitro* assays and xenograft tumor model experiments was made using a two‐sided Student's *t*‐test. Differences were considered statistically significant when *P* value was less than 0.05. The number of experiments performed is indicated in the corresponding figure legend.

## 3 Results

### 3.1 Dasatinib prevents NRG‐induced MMP13 upregulation

**Figure 1:**
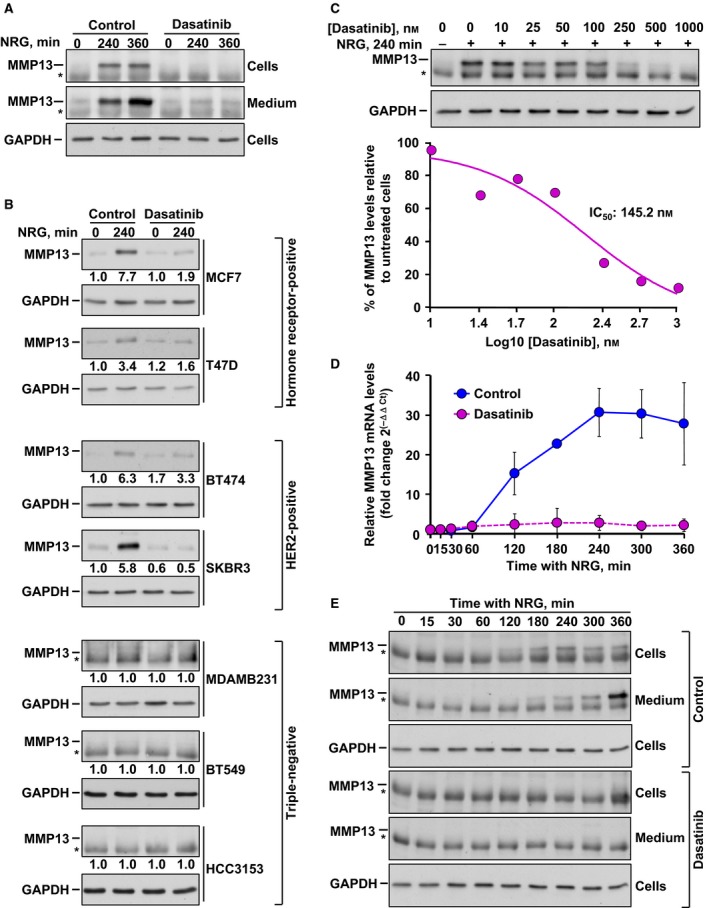
Dasatinib prevents NRG‐induced MMP13 upregulation. (A) Western blotting analysis of the effect of dasatinib on NRG‐induced MMP13 upregulation in MCF7 cell extracts and cell culture medium. MMP13 was immunoprecipitated from both cell extracts and culture medium at the indicated times. The asterisks indicate the heavy chain band of the antibody used for MMP13 immunoprecipitation. Levels of GAPDH were used as a loading control. (B) Effect of dasatinib on NRG‐induced MMP13 protein levels in different breast cancer cell lines analyzed by western blotting. The numbers shown below the blots indicate a quantitative measurement of the fold change in MMP13 with respect to unstimulated cells. The asterisks indicate the heavy chain band of the antibody used for MMP13 immunoprecipitation. GAPDH levels were used as a loading control. (C) Analysis of the dose–response effect of dasatinib on MMP13 protein levels in MCF7 cells by western blotting. Seven different concentrations (ranging from 10 to 1000 nm) were used. The asterisk indicates the heavy chain band of the antibody used for MMP13 immunoprecipitation. Levels of GAPDH were used as a loading control. The graphic to the bottom represents the IC_50_ determination of the inhibition of MMP13. (D) Quantitative RT‐PCR detection of MMP13 mRNA levels in NRG‐stimulated MCF7 cells pretreated or not with dasatinib. MMP13 mRNA levels were normalized against that of GAPDH and relativized to those from unstimulated cells. The graph represents the mean ± SD of data from two independent experiments. (E) Time course of the effect of dasatinib on NRG‐induced MMP13 upregulation in MCF7 cell extracts and cell culture medium analyzed by western blotting. The asterisks indicate the heavy chain band of the antibody used for MMP13 immunoprecipitation. GAPDH was used as a loading control. Data information: In (A–C) and (E), results from a representative experiment that was repeated twice are shown.

Former reports indicated that the clinically approved tyrosine kinase inhibitor dasatinib prevented activation of the ERK1/2 route in breast cancer cells overexpressing HER2 (Seoane *et al*., 2010). This fact, together with the important role of certain cellular targets of dasatinib in the regulation of the dissemination and seeding of breast cancer cells (Zhang *et al*., 2013), led us to explore the effect of this kinase inhibitor in NRG‐induced MMP13 upregulation. To that end, we first explored the action of dasatinib using MCF7 cells, which express near normal levels of HER2 and other HER receptors and respond mitogenically to the addition of exogenous soluble NRG (Holmes *et al*., 1992). As previously reported (Seoane *et al*., 2016), addition of NRG augmented MMP13 in MCF7 cell lysates and this upregulation was accompanied by the accumulation of MMP13 in the culture media (Fig. 1A). Preincubation of MCF7 cells with dasatinib prevented such upregulation (Figs 1A and S1). We next explored the action of dasatinib on NRG‐induced upregulation of MMP13 levels using several cell lines belonging to different breast cancer subtypes. Addition of NRG increased the amount of MMP13 in hormone receptor‐positive cellular models (MCF7 and T47D cells), as well as in HER2‐overexpressing cellular models (BT474 and SKBR3 cells), and such effect was prevented by dasatinib (Fig. 1B). NRG failed to increase MMP13 production in the triple‐negative cell lines. The effect of dasatinib was dose dependent, reaching complete inhibition of MMP13 upregulation at a dose of 1 μm dasatinib and with an IC_50_ value of 145.2 nm (Fig. 1C).

To analyze whether dasatinib affected the transcription of the mRNA coding for MMP13, MCF7 cells were preincubated with the drug and then stimulated or not with NRG for different times. NRG treatment caused upregulation of the mRNA coding for MMP13 (Fig. 1D). The effect of NRG was already detectable at 120 min of treatment and reached a peak at 240 min. Preincubation of MCF7 cells with dasatinib prevented NRG‐induced MMP13 transcription (Fig. 1D). Analyses of the effect of the drug on NRG‐induced MMP13 protein accumulation in cell lysates or in culture media were consistent with the qRT‐PCR data, although the effect of NRG on the metalloprotease in the media was accumulative over time (Fig. 1E).

### 3.2 Action of dasatinib on signaling by the NRG–HER system

**Figure 2:**
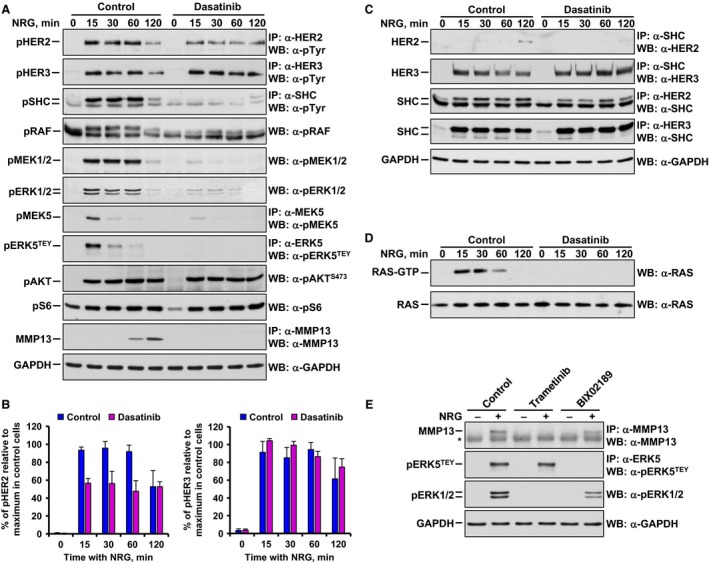
Action of dasatinib on the NRG–HER signaling system in MCF7 cells. (A) Western blotting analysis showing the time‐course effect of dasatinib on the phosphorylation of HER receptors and downstream proteins involved in NRG signaling. In addition, the effect of dasatinib in NRG‐induced MMP13 upregulation was detected. Levels of GAPDH were used as a loading control. (B) Graphical representation of the quantification of HER2 and HER3 phosphorylation after NRG stimulation in MCF7 cells pretreated or not with dasatinib. Data were relativized to maximal phosphorylation in untreated cells and are expressed as the mean ± SD of three independent experiments performed as in (A). (C) Time‐course effect of dasatinib on the interaction of SHC with HER2 and HER3 after NRG stimulation by western blotting. Protein–protein interaction was analyzed by co‐immunoprecipitation. GAPDH levels were used as a loading control. (D) Effect of dasatinib on the time course of RAS activation induced by NRG analyzed by western blotting. A RAS‐GTP pull‐down assay was performed to measure the levels of activation of RAS. Levels of total RAS were used as a loading control. (E) Analysis of the effect of 1 μm trametinib or 10 μm BIX02189 on the regulation of MMP13, pERK5^TEY^, and pERK1/2 levels after NRG stimulation (15 min for pERK5 ^TEY^ and pERK1/2 or 4 h for MMP13) by western blotting. The asterisk indicates the heavy chain band of the antibody used for MMP13 immunoprecipitation. Levels of GAPDH were used as a loading control. Data information: Experiments were repeated three times with similar results. Representative results of all the findings are shown.

To explore how dasatinib inhibited NRG‐induced upregulation of MMP13, NRG was added for different times to MCF7 cells, which were preincubated or not with dasatinib. Subsequently, the activation status of different proteins involved in NRG signaling was analyzed by western blotting by measuring their phosphorylation levels. Dasatinib treatment did not affect the activation of HER3 induced by NRG, but it exerted a partial inhibitory effect on the tyrosine phosphorylation status of HER2 (Fig. 2A,B). Dasatinib inhibited the phosphorylation of the adaptor protein SHC (Fig. 2A). Therefore, the possibility that dasatinib could inhibit interaction of SHC proteins with HER receptors was explored by coprecipitation experiments. Treatment of MCF7 cells with NRG provoked association of SHC with HER3 (Fig. 2C). The amount of SHC that interacted with HER2 was much lower. Preincubation of MCF7 cells with dasatinib did not affect the interaction of HER3 with SHC. Therefore, while dasatinib exerted a profound inhibitory effect of NRG‐induced SHC tyrosine phosphorylation, the drug did not apparently affect the ability of SHC to interact with HER3 and poorly affected interaction with HER2.

Dasatinib treatment inhibited the activation of RAS as indicated by a RAS‐GTP pull‐down assay (Fig. 2D). Consequently, activation of RAF, MEK1/2, and ERK1/2, analyzed by the detection of their phosphorylated forms, was inhibited by dasatinib (Fig. 2A). In addition to the RAS‐RAF‐MEK‐ERK1/2 route, NRGs activate the PI3K and the ERK5 pathways (Esparís‐Ogando *et al*., 2002; Montero *et al*., 2011b; Seoane *et al*., 2016). Preincubation of MCF7 cells with dasatinib strongly inhibited phosphorylation of ERK5 at its TEY microactivation domain (Fig. 2A). Moreover, dasatinib prevented phosphorylation of MEK5, which acts as the ERK5 upstream activating kinase (Fig. 2A). In contrast, NRG‐induced activation of S6 and AKT, which were used as readouts of activation of the mTORC1 and mTORC2 pathways, respectively, was not affected by preincubation with dasatinib (Fig. 2A). These results indicated that the PI3K route does not have a major role in MMP13 upregulation upon activation of NRG receptors.

The above data suggested that dasatinib affected NRG–MMP13 signaling likely by inhibiting the ERK kinases. To explore the contribution of the ERK routes to the regulation of MMP13 levels by NRG, agents that target these kinases were used. Preincubation of MCF7 cells with the ERK1/2 route inhibitor trametinib (Gilmartin *et al*., 2011), which acts on the ERK1/2 upstream activating kinases, inhibited NRG‐induced MMP13 upregulation (Fig. 2E). Western blotting of pERK1/2 demonstrated the inhibitory effect of trametinib on this route, sparing the ERK5 pathway. On the other side, preincubation with the ERK5 pathway inhibitor BIX02189, which inhibits MEK5 (Tatake *et al*., 2008), had a substantially smaller effect on NRG‐induced regulation of MMP13 levels, even though the drug completely inhibited the activation of ERK5 caused by NRG (Fig. 2E). These data suggest a predominant role of the ERK1/2 route over the ERK5 pathway as intermediates in NRG‐induced upregulation of MMP13, and they reveal that only dasatinib exerts a dual inhibitory effect on the ERK1/2 and ERK5 routes.

### 3.3 Identification of dasatinib‐binding proteins in MCF7 cells

**Figure 3:**
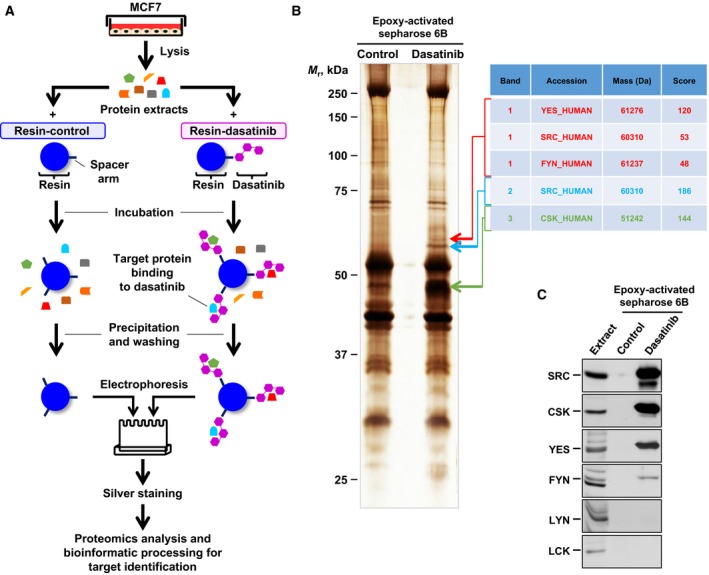
Identification of dasatinib‐binding proteins in MCF7 cells. (A) Schematic representation of the chemical proteomics approach used for dasatinib‐binding protein identification in MCF7 cells. (B) Silver‐stained SDS/PAGE of protein–resin complexes. MCF7 cell lysates were incubated with dasatinib coupled to epoxy‐activated Sepharose 6B medium (dasatinib‐resin) or to uncoupled epoxy‐activated Sepharose 6B medium (control resin). Bands of interest were excised from the gel, and the proteins from these bands were analyzed by MALDI‐TOF. Main proteins identified in bands 1–3 across Swiss‐Prot database are shown in the table to the right, together with the proteins' molecular weight and the proteins' score Mascot reported. *M*_*r*_ refers to molecular mass (kDa). (C) Western blotting analysis of the interaction of SRC, CSK, YES, FYN, LYN, and LCK with dasatinib by pull‐down experiments with the resins mentioned in (B). Cell extracts were used to detect total protein levels. Data information: In (B,C), results from a representative experiment that was repeated twice are shown.

To further explore the mechanism by which dasatinib exerted its inhibitory action on the control of NRG‐induced MMP13 upregulation, we next attempted to identify potential targets of dasatinib in MCF7 cells using a chemical proteomics approach (Fig. 3A). Dasatinib was immobilized on epoxy‐activated Sepharose medium, and the resultant resin was used as an affinity reagent to identify binding proteins in MCF7 cells. As a nonspecific binding control, MCF7 cell lysates were incubated with uncoupled epoxy‐activated Sepharose medium. Bound proteins were resolved by SDS/PAGE followed by silver staining. Bands corresponding to proteins specifically interacting with the dasatinib‐coupled resin were excised from the gel, and the proteins from these bands were analyzed by MALDI‐TOF. Three bands of apparent molecular weights of 65, 63, and 40 kDa were observed to specifically interact with the dasatinib‐containing resin (Fig. 3B). The 65‐kDa band included YES, SRC, and FYN. The 63‐kDa band included SRC, and the 40‐kDa band contained CSK.

These experiments identified SRC, CSK, YES, and FYN as the most prominent interactors with the dasatinib‐containing resin (Fig. 3B). To validate the identification of these kinases as proteins retained by the dasatinib‐coupled resin, pull‐down experiments followed by western blotting were performed. SRC, CSK, YES, and FYN were bound to the dasatinib‐containing resin, but not to the control resin that did not contain dasatinib (Fig. 3C). The amount of those proteins bound to the dasatinib‐containing resin was substantial, except for FYN. No detectable binding of LYN and LCK to immobilized dasatinib was observed (Fig. 3C), in agreement with the lack of identification of these proteins in the MALDI‐TOF experiments (Fig. 3B and data not shown). The above results suggested that SRC, YES, and FYN, but not LYN or LCK, were the potential targets of dasatinib in MCF7 cells. It is relevant to discuss that even though CSK can bind dasatinib (Karaman *et al*., 2008; Rix *et al*., 2007), whether CSK was indirectly retained on the resin because of its reported interaction with SRC family members (Vielreicher *et al*., 2007) could not be excluded from the experimental data obtained.

### 3.4 Participation of SRC family kinases in NRG‐induced MMP13 upregulation

**Figure 4:**
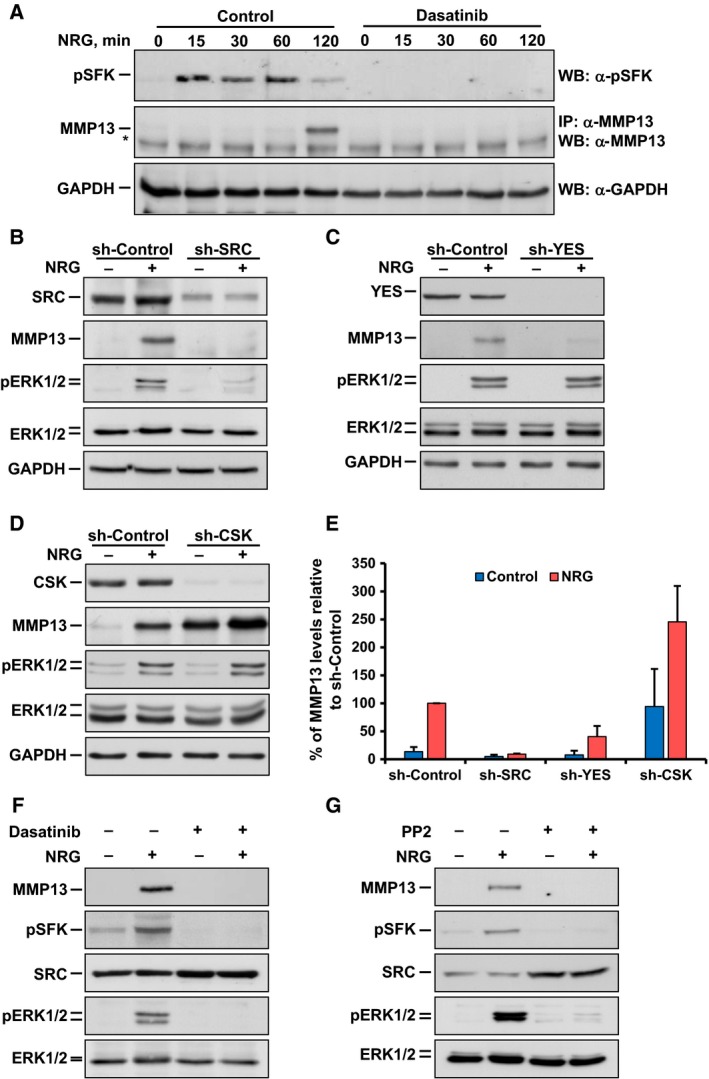
Participation of SRC family kinases in NRG‐induced MMP13 upregulation. (A) Time course of the effect of dasatinib on SRC family kinases (SFKs) phosphorylation after NRG stimulation in MCF7 cells analyzed by western blotting. In addition, the effect of dasatinib on NRG‐induced MMP13 upregulation was analyzed. The asterisk indicates the heavy chain band of the antibody used for MMP13 immunoprecipitation. Levels of GAPDH were used as a loading control. (B–D), Western blotting analysis of the effect of the knockdown of SRC (B), YES (C), and CSK (D) on the regulation of MMP13 and pERK1/2 after NRG stimulation (15 min for pERK1/2 and ERK1/2 or 4 h for MMP13) in MCF7 cells. Cells were infected with lentivirus containing the shRNA control (sh‐Control) or the shRNA sequence targeting SRC, YES, or CSK. To verify the knockdown efficiency, target protein levels were analyzed. GAPDH levels were used as a loading control. (E) Bar graph representing the quantification of the effect of the knockdown of SRC, YES, and CSK on MMP13 protein levels in NRG‐stimulated MCF7 cells. Data were relativized to those levels in NRG‐stimulated cells infected with sh‐Control and are expressed as the mean ± SD of two independent experiments performed as in (B–D). (F, G) Analysis of the comparative effect of 1 μm dasatinib and 10 μm PP2 on the regulation of MMP13, pSFKs, and pERK1/2 after NRG stimulation (15 min for pSFKs, pERK1/2 or 4 h for MMP13) in MCF7 cells by western blotting. Levels of ERK1/2 were used as a loading control. Data information: Experiments were repeated twice with similar results. Representative results of all the findings are shown.

We next explored the contribution of dasatinib‐interacting proteins of the SRC family of kinases (SFKs) as intermediates in the control of MMP13 production upon activation of NRG receptors. First, the effect of dasatinib on the activation of SFKs was explored. To this end, a phospho‐antibody that interacts with active forms of several SFKs, including SRC, YES, and FYN, was used. Treatment of MCF7 cells with NRG caused upregulation of pSFKs (Fig. 4A). Preincubation of MCF7 cells with dasatinib completely inhibited NRG‐induced phosphorylation of SFKs at the region recognized by this antibody.

As dasatinib may act on several SFKs, the drug cannot be used to define the individual contribution of each of them to NRG‐induced MMP13 upregulation. To address this question, shRNA lentiviral vectors were used to knockdown the expression of SFKs. From the five shRNA lentiviral vectors used for each protein, at least two of them caused substantial knockdown of SRC, YES, or CSK and generated analogous results, although data with only one shRNA sequence are presented (Fig. 4B–E and data not shown). Analysis of the participation of FYN was not performed because of the low interaction of this protein with the dasatinib‐coupled resin (Fig. 3B,C). Knockdown of SRC inhibited the upregulation of MMP13 upon stimulation of MCF7 cells with NRG (Fig. 4B,E). In addition, knockdown of SRC also reduced NRG‐induced upregulation of pERK1/2 (Fig. 4B). Knockdown of YES also inhibited NRG‐induced MMP13 upregulation in MCF7 cells (Fig. 4C,E), but was less efficient than the knockdown of SRC (Fig. 4E). Knockdown of YES did not appreciably affect NRG‐induced upregulation of pERK1/2 (Fig. 4C). Knockdown of CSK, which acts as a kinase that inhibits SFKs by interacting with their C‐terminal region (Okada, 2012), resulted by itself in upregulation of MMP13 levels (Fig. 4D,E). Interestingly, CSK knockdown did not affect pERK1/2 levels when compared to those present in control vector‐infected MCF7 cells (Fig. 4D). Addition of NRG caused an additional increase in MMP13 in CSK‐knockdown cells (Fig. 4D,E). Finally, we compared the effectiveness of dasatinib to that of PP2, an alternative SFKs inhibitor (Hanke *et al*., 1996). Preincubation of MCF7 cells with PP2 had a similar effect to that of dasatinib on NRG‐induced upregulation of MMP13 (Fig. 4F,G). Moreover, PP2 reduced pERK1/2 levels, as occurred with dasatinib.

### 3.5 Action of dasatinib on NRG‐induced biological responses

**Figure 5:**
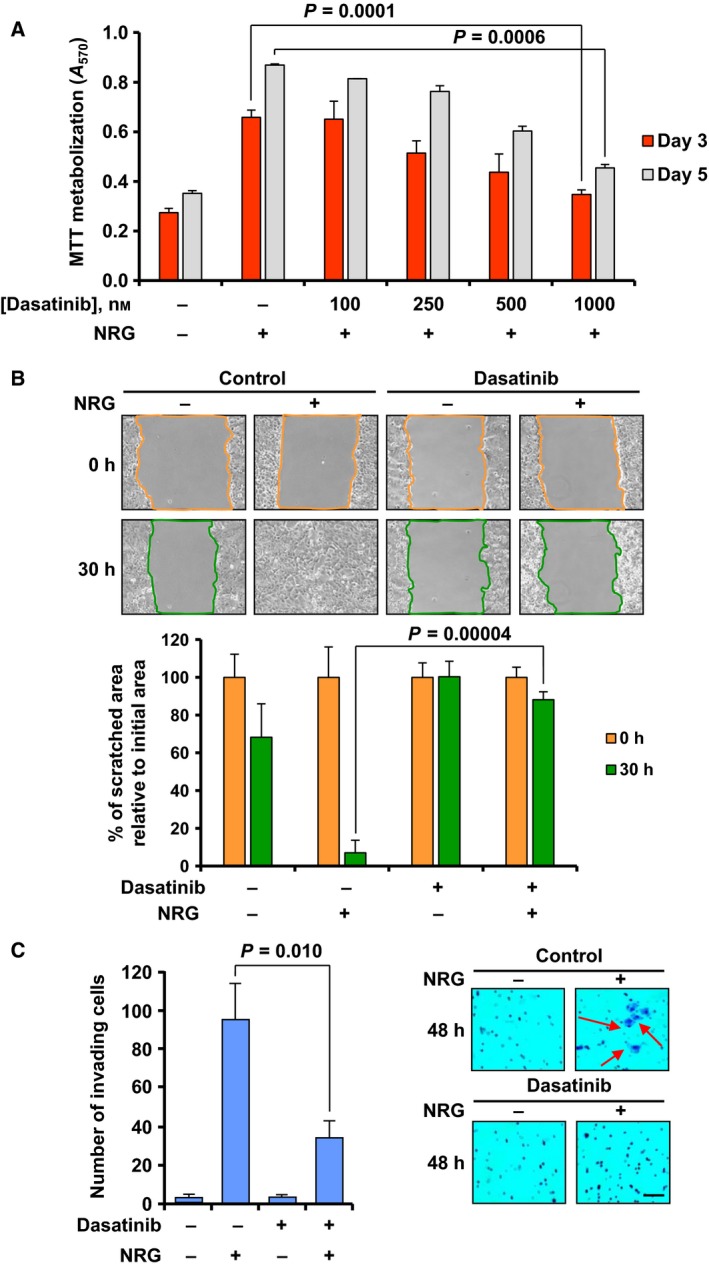
Dasatinib inhibits NRG‐induced proliferation, migration, and invasion. (A) Bar graph representing the dose–response effect of dasatinib on NRG‐induced proliferation in MCF7 cells. Cell proliferation was determined by MTT metabolization 3 and 5 days after NRG stimulation. Four different concentrations (ranging from 100 to 1000 nm) were used. (B) Representative images from the wound healing assay showing the effect of dasatinib on NRG‐induced migration of MCF7 cells at 0 and 30 h after NRG stimulation. The colored lines delimit the area of the wounded region. The bar graph at the bottom represents the quantitation of the wounded area with respect to the initial area in each condition (0 h). (C) Bar graph showing the number of MCF7 invading cells 48 h after NRG stimulation and the effect of pretreatment with dasatinib. Cells that were able to pass through the Matrigel layer were fixed, stained with crystal violet, and counted. Representative images of the experiment are shown on the right. The arrows point to cells stained with crystal violet. Scale bar = 100 μm. Data information: Results are presented as the mean ± SD of triplicate of an experiment that was repeated three times. Comparisons of means between two independent groups were made using a two‐sided Student's *t*‐test.

Activation of NRG receptors facilitates biological functions associated with a pro‐oncogenic phenotype, including stimulation of cell proliferation, migration, and local spreading, or promotion of metastatic dissemination (Montero *et al*., 2008). To determine whether dasatinib affected NRG‐induced proliferation, MCF7 cells incubated with different concentrations of the drug were then stimulated with NRG for 3 and 5 days. Dasatinib inhibited NRG‐induced proliferation of MCF7 cells in a dose‐dependent manner (Fig. 5A). Whether dasatinib could also affect NRG‐induced migration and invasion was also explored. For the migration assay, MCF7 cells were allowed to reach confluence, and then, the surface of the monolayers was scratched with a pipette tip to create an area without cells. Then, cells were exposed to dasatinib for 3 h before the addition of NRG, and photographs of the wounded regions of the dishes were obtained every 2 h. Movies were also recorded. Dasatinib inhibited NRG‐induced migration of MCF7 cells (Fig. 5B and Movies [Link], [Link], [Link], [Link]). For the invasion assay, MCF7 cells treated with dasatinib for 3 h were then stimulated with or without NRG for 48 h. As shown in Fig. 5C, dasatinib treatment inhibited NRG‐induced cell invasion, as assessed by a transwell assay.

**Figure 6:**
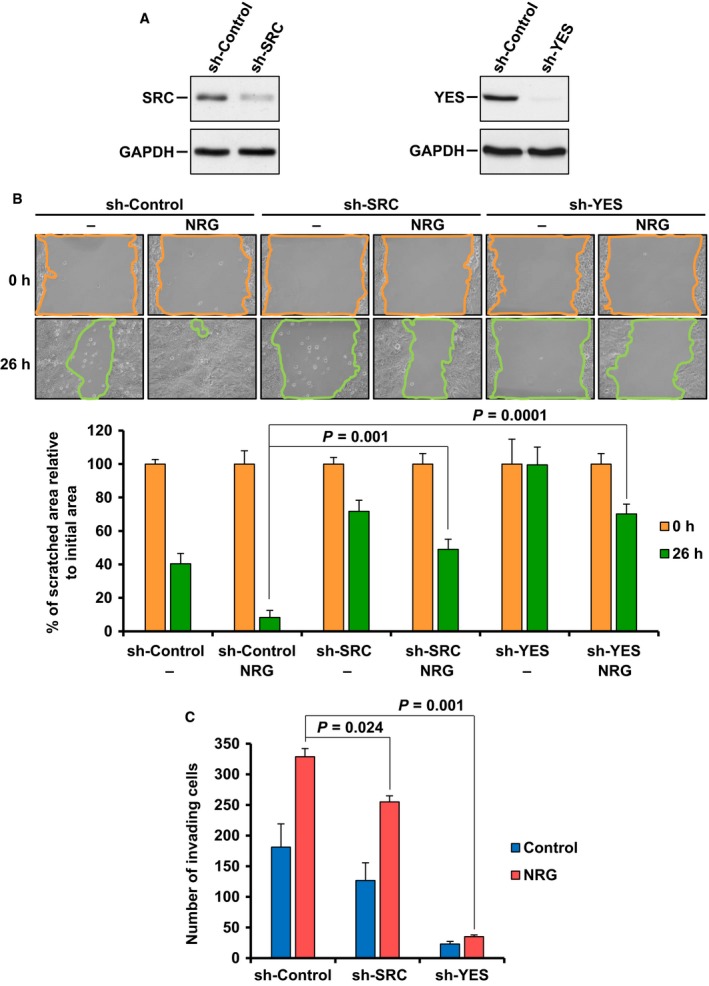
Effect of the knockdown of SRC and YES on NRG‐induced migration and invasion. (A) Western blotting analysis of the knockdown of SRC and YES in MCF7 cells. GAPDH levels were used as a loading control. (B) Representative images from the wound healing assay showing the effect of the knockdown of SRC and YES on NRG‐induced migration of MCF7 cells at 0 and 26 h after NRG stimulation. The bar graph at the bottom represents the quantitation of the wounded area with respect to the initial area in each condition (0 h). (C) Bar graph showing the number of invading cells 72 h after NRG stimulation. Data information: Results are presented as the mean ± SD of triplicate of an experiment that was repeated three times. Comparisons of means between two independent groups were made using a two‐sided Student's *t*‐test.

As SRC and YES are intermediates in the control of MMP13 production upon activation of NRG receptors, and dasatinib suppressed NRG‐induced migration and invasion, whether the knockdown of SRC or YES had the same antimetastatic properties was investigated. To this end, the above‐mentioned shRNA lentiviral vectors were used to knockdown the expression of SRC or YES in MCF7 cells (Fig. 6A), and wound healing and invasion assays were performed. Both knockdowns inhibited migration and invasion after NRG stimulation in MCF7 cells (Fig. 6B,C).

We also explored whether the NRG–MMP13 signaling axis could have a role in the control of cell dissemination in other breast cancer cellular models. BT474 cells were chosen to carry out wound healing and invasion assays as they showed the highest MMP13 induction after NRG stimulation among all the cell lines included in our studies (Fig. 1B). Dasatinib inhibited both NRG‐induced migration and invasion of BT474 cells (Fig. S2A,B).

**Figure 7:**
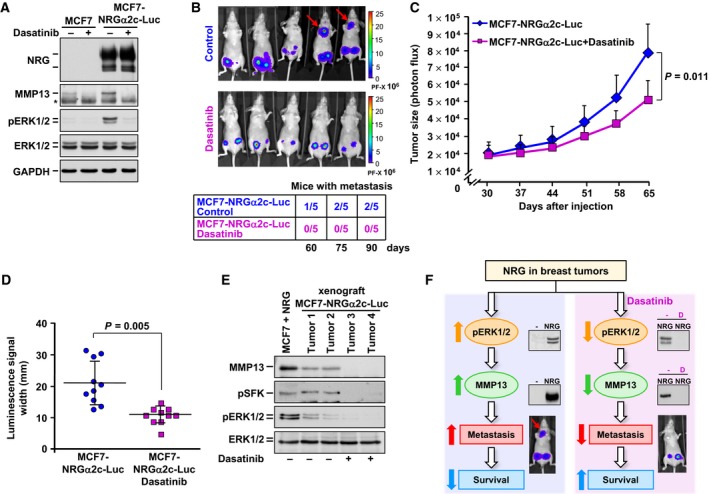
Dasatinib inhibits cell proliferation, local dissemination, and metastatic dissemination *in vivo*. (A) Analysis of NRG, MMP13, pERK1/2, and ERK1/2 in MCF7 and MCF7‐NRGα2c‐Luc cells pretreated or not with dasatinib for 24 h by western blotting. The asterisk indicates the heavy chain band of the antibody used for MMP13 immunoprecipitation. Levels of GAPDH were used as a loading control. (B) Bioluminescence whole‐body images of mice injected with MCF7‐NRGα2c‐Luc cells and treated with dasatinib or not (vehicle control). Images were taken 90 days after injection. The rainbow‐colored bar at the right of the images represents the intensity scale of the luminescence signal. The arrows point to the disseminated tumoral masses. The table at the bottom shows the number of mice with metastasis in each experimental group at 60, 75, and 90 days after injection (*n *=* *5 mice per experimental condition). (C) Graphic representation of primary tumor growth analyzed weekly by measuring photon flux with an IVIS 50 Imaging system after the beginning of the treatment in each group. The treatment began 30 days after injection. Data are presented as the mean ± SD of the primary tumors assayed in each experimental condition. Comparisons of means between two independent groups were made using a two‐sided Student's *t‐*test. (D) Column scatter graph showing the local dissemination of the primary tumors in each group as indicated by measurements of the width of the luminescent signals 90 days after injection. Horizontal bars in each plot indicate the location of the mean. Data are presented as the mean ± SD of the primary tumors of each group. Comparisons of means between two independent groups were made using a two‐sided Student's *t‐*test. (E) Western blotting analysis of MMP13, pSFK, and pERK1/2 in primary tumor samples from MCF7‐NRGα2c‐Luc cell‐injected mice treated with dasatinib (tumors 3 and 4) or not (tumors 1 and 2) 90 days after injection. NRG‐stimulated MCF7 cell extracts were used as a control of detection of the above‐mentioned proteins. Levels of ERK1/2 were used as a loading control. (F) Schematic representation of the effect of NRG on MMP13 upregulation and breast cancer metastasis, as well as the expected effect of dasatinib on this process. The letter D refers to dasatinib. Data information: In (A), results from a representative experiment that was repeated twice are shown.

To evaluate the *in vivo* capability of dasatinib to affect the above‐explored biological functions upon activation of NRG receptors, MCF7 cells expressing the NRG isoform NRGα2c and the luciferase gene (MCF7‐NRGα2c‐Luc cells) were used. MCF7‐NRGα2c‐Luc cells constitutively expressed MMP13 and pERK1/2 at levels higher than parental MCF7 cells (Fig. 7A). Treatment with dasatinib decreased both MMP13 and pERK1/2 levels (Fig. 7A). When these cells were injected into the mammary fat pad of nude mice, at two sites per mouse, tumors derived from untreated mice were larger than those generated in mice treated with dasatinib, indicating that the drug reduced tumor growth (Fig. 7B,C). Dasatinib treatment also reduced local spreading compared to untreated tumors, as indicated by measurements of the width of the luminescent signals (Fig. 7D). In mice injected with MCF7‐NRGα2c‐Luc cells, some tumors had a tendency to expand to new sites beyond the site of implantation of the cells (Fig. 7B). Such dissemination of MCF7‐NRGα2c‐Luc cells was not observed in animals treated with dasatinib (Fig. 7B). Western blotting analyses of tumor samples obtained from untreated mice indicated that tumors originating from MCF7‐NRGα2c‐Luc cells expressed MMP13, pSFKs, and pERK1/2 (Fig. 7E). In mice treated with dasatinib, the drug decreased the levels of MMP13 as well as the phosphorylated forms of SFKs and ERK1/2 (Fig. 7E). Taken together, the above data demonstrated that dasatinib inhibited several basilar pro‐oncogenic responses triggered upon activation of the NRG receptors, including metastatic dissemination.

## 4 Discussion

Metastatic dissemination constitutes a critical pathophysiological condition in breast cancer that may determine a fatal outcome of this disease. For this reason, intense efforts are being made to define the mechanisms responsible for such dissemination, in order to find strategies to prevent or treat metastases (Obenauf and Massagué, 2015). Studies from our group indicated that expression of NRGs in breast tumors was linked to poor patient outcome as a consequence of higher metastatic dissemination properties of tumors expressing these HER ligands (Seoane *et al*., 2016). Transcriptomic as well as functional studies pointed to MMP13 as a relevant mediator of NRG‐induced metastatic dissemination. The present study was initiated with the purpose of finding a drug which could prevent NRG‐induced upregulation of MMP13, focusing on approved drugs because they may be more suitable for clinical development.

Several precedents pointed to dasatinib as an attractive drug candidate. In fact, this drug may reduce metastatic spreading of different types of tumors (Montero *et al*., 2011a; Zhang *et al*., 2009). Furthermore, dasatinib has been shown to decrease the amount of pERK1/2 kinases (Seoane *et al*., 2010), which participate in a pathway that promotes MMP13 production (Seoane *et al*., 2016). Here we show that dasatinib reduced MMP13 upregulation and pERK1/2 activation upon stimulation of NRG receptors. In addition, dasatinib reduced several prometastatic properties of MCF7 cells such as migration or invasion.

Systematic analyses of the major signaling pathways activated by NRG receptors indicated that dasatinib had a marginal effect on HER receptor tyrosine phosphorylation. In fact, NRG‐induced HER3 tyrosine phosphorylation was unaffected by treatment with dasatinib, while HER2 tyrosine phosphorylation was marginally inhibited. In contrast to these marginal effects on NRG‐induced receptor phosphorylation, the drug exerted a strong inhibitory effect on proximal signaling downstream of receptor tyrosine phosphorylation. In fact, while the interaction of HER3 receptors with the adaptor protein SHC was unaffected, phosphorylation of SHC was compromised in cells treated with the drug. The different preferences in the interactions between HER2 and HER3 receptors and SHC isoforms have not been reported previously and need to be explored further. As a consequence of inhibition of SHC activation, RAS activation, as well as RAF, MEK, and ERK1/2 activating phosphorylations, was also profoundly affected in cells treated with dasatinib. In addition, dasatinib strongly reduced the activation of ERK5 and its upstream activating kinase MEK5. Experiments using inhibitors of the ERK1/2 and ERK5 pathways demonstrated the effectiveness of ERK1/2 pathway inhibitors in preventing NRG‐induced MMP13 upregulation. Therefore, reduction in the RAS‐RAF‐MEK1/2‐ERK1/2 route appears to be responsible for the inhibitory effect of dasatinib on the neosynthesis of MMP13 upon activation of NRG receptors. Of note, no effect of dasatinib was found when pAKT and pS6 were evaluated, indicating that this route is neither affected by the drug nor involved in NRG‐induced upregulation of MMP13. Indirectly, the fact that stimulation of the PI3K/AKT/mTOR pathway by NRG is not inhibited by dasatinib also supports the concept that very proximal signaling, that is, ligand binding, receptor dimerization, and transautophosphorylation, is unaffected by the drug.

Dasatinib has been shown to affect the tyrosine kinase activity of several receptor as well as nonreceptor tyrosine kinases (Karaman *et al*., 2008; Montero *et al*., 2011a). To define the targets of dasatinib, we used a chemical proteomics approach. These studies indicated that dasatinib could potentially act through SRC, YES, CSK, and FYN, which have been described among the most sensitive targets of dasatinib (Karaman *et al*., 2008; Montero *et al*., 2011a). SRC has formerly been implicated in metastatic dissemination in breast cancer (Zhang *et al*., 2009, 2013). Moreover, SRC may associate with HER receptors (Kim *et al*., 2005), and previous data in HER2‐overexpressing breast cancer cells indicated that SRC inhibition prevented ERK1/2 activation (Seoane *et al*., 2010). We observed that knockdown of SRC reduced ERK1/2 activation and MMP13 upregulation upon stimulation of NRG receptors in MCF7 cells. In addition, knockdown of YES also reduced MMP13 expression in MCF7 cells. However, knockdown of YES had a smaller inhibitory effect on NRG‐induced MMP13 upregulation than knockdown of SRC. Moreover, knockdown of YES did not affect NRG‐induced upregulation of pERK1/2. This is particularly relevant as the ERK1/2 route appeared to have a major role in controlling MMP13 production (Seoane *et al*., 2016). However, the YES‐knockdown data, together with data obtained from the knockdown of CSK, strongly suggest that routes other than the ERK1/2 pathway may control MMP13 production. In fact, knockdown of CSK *per se* caused a substantial increase in the production of MMP13. Moreover, such increase in MMP13 caused by CSK knockdown was not accompanied by a parallel increase in ERK1/2 activation, indicating that knockdown of CSK likely acted through a pERK1/2‐independent route. Therefore, our data indicate that ERK kinases may play a substantial role in NRG‐induced upregulation of MMP13 production. However, the effect of CSK knockdown on pERK1/2 and MMP13 production also demonstrates that control of MMP13 production is complex, being regulated by ERK1/2‐dependent as well as ERK1/2‐independent pathways. Definition of these yet unknown routes will require additional studies.

Neuregulin expression has been linked to poor patient outcome in breast cancer, probably by facilitating dissemination of breast cancer cells to lymph nodes (Seoane *et al*., 2016). In the present study, we intended to model this clinical situation by using breast cancer cells engineered to constitutively express NRG. When these human breast cancer cells expressing NRG were injected in mice, they created tumors that spread locally and disseminated to sites distant from the place of tumor cell injection. Dasatinib had an inhibitory action on these biological properties of the NRG‐expressing breast cancer cells. Moreover, dasatinib reduced tumor growth and local spreading. This finding is quite relevant, not only from the biological point of view, but also from the therapeutic side. In fact, dasatinib is approved for use in human diseases such as chronic myelogenous leukemia. The fact that dasatinib is licensed for use in humans opens the possibility of incorporating this drug to the armamentarium to fight breast cancer dissemination, especially in tumors fed by NRG (Fig. 7F), which may occur in up to 50% of breast tumors (de Alava *et al*., 2007). At present, a clinical trial in metastatic breast cancer using dasatinib is ongoing, and the phase Ib results will be soon reported. If dasatinib results effective in this clinical setting, it will be important to develop its use in other oncological entities in which the NRG–MMP13 signaling axis may play a relevant role in the control of tumor cell dissemination.

## 5 Conclusions

In summary, in the current study, we have shown that dasatinib restricts NRG‐induced MMP13 upregulation and metastatic dissemination of breast cancer cells. In addition, SRC family kinases were found to be the main cellular targets of dasatinib. Knockdown of SRC or YES strongly inhibited NRG‐induced MMP13 upregulation. Dasatinib treatment or knockdown of SRC also inhibited ERK1/2 kinases, which were required for NRG‐induced MMP13 upregulation. As dasatinib is a drug already approved in the cancer clinic, the identification that the prometastatic NRG–MMP13 axis is sensitive to dasatinib opens the possibility of incorporating this drug to the armamentarium to fight breast cancer metastatic dissemination. Moreover, these studies may have a wider impact, as it is likely that other tumors may use MMP13 production to facilitate tumor cell dissemination.

## Author contributions

AOR, SS, AO, and JCM performed experiments, prepared figures, and wrote parts of the manuscript. AP supervised research and wrote parts of the manuscript. All authors corrected and approved the final version of the manuscript.

## Supporting information


**Fig. S1.** Dasatinib prevents NRG‐induced MMP13 up‐regulation at long‐term.

**Fig. S2.** Dasatinib inhibits NRG‐induced migration and invasion in BT474 cells.

**Movie S1.** Migration assay of unstimulated MCF7 cells.

**Movie S2.** Migration assay of MCF7 cells stimulated with NRG.

**Movie S3.** Migration assay of MCF7 cells pretreated with dasatinib and unstimulated with NRG.

**Movie S4.** Migration assay of MCF7 cells pretreated with dasatinib and stimulated with NRG.
