# Supplementary figures and images for: Regulation of the prometastatic neuregulin–MMP13 axis by SRC family kinases: therapeutic implications
Source: Mol Oncol. 2017 Oct 31;11(12):1788–805. doi: 10.1002/1878-0261.12145 (PMC5709617; doi:10.1002/1878-0261.12145)

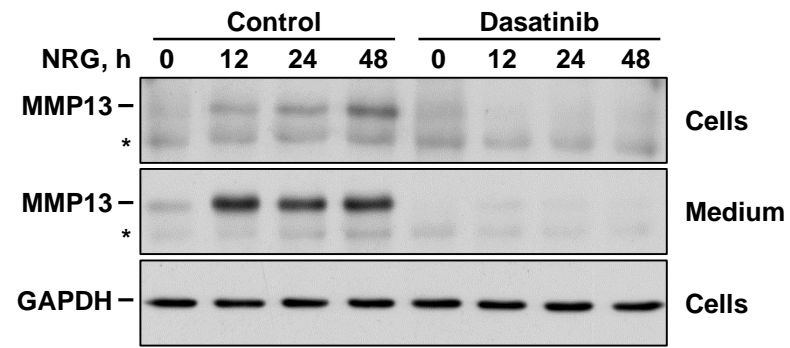

**Supplementary Figure S1**

Supplement: Supplementary file 1 — Fig. S1. Dasatinib prevents NRG‐induced MMP13 up‐regulation at long‐term. [file MOL2-11-1788-s001.pdf]

**A**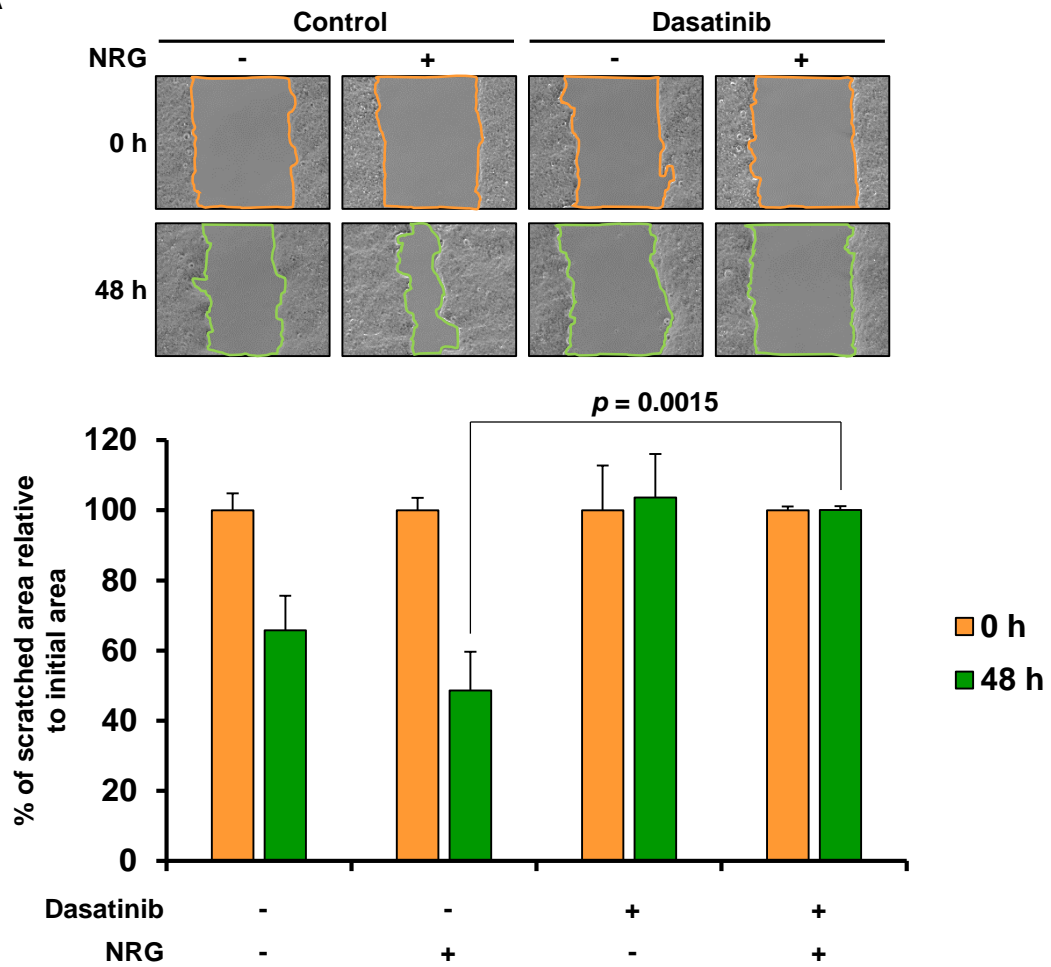**B**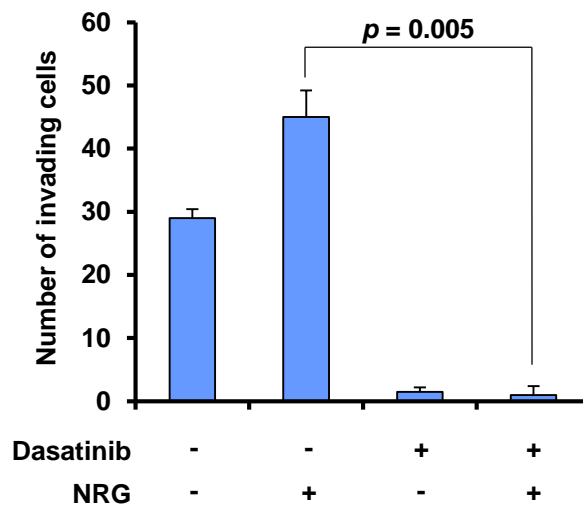**Supplementary Figure S2**

Supplement: Supplementary file 2 — Fig. S2. Dasatinib inhibits NRG‐induced migration and invasion in BT474 cells. [file MOL2-11-1788-s002.pdf]
